# Feasibility study of the internet-based intervention ‘Strategies for Empowering activities in Everyday life’ (SEE 2.0) for use by people with chronic diseases and long-term disorders in healthcare: a study protocol

**DOI:** 10.1136/bmjopen-2025-102026

**Published:** 2025-10-21

**Authors:** Maria Larsson-Lund, Johanna Karlsson Sundbaum, Eva Mansson Lexell, Linda Spinord

**Affiliations:** 1 Department of Health, Education and Technology, Luleå tekniska universitet, Luleå, Sweden; 2 Department of Public Health and Clinical Medicine, Umeå University, Umeå, Sweden; 3 Department of Health Sciences, Lund University, Lund, Sweden; 4 Department of Neurology and Rehabilitation Medicine, Memory disorders and Geriatrics, Skåne University Hospital, Lund, Sweden

**Keywords:** Self-Management, Chronic Disease, eHealth, Telemedicine

## Abstract

**Introduction:**

Proactive and easily accessible interventions are needed to support people with long-term health conditions who experience limitations in activities of everyday life. However, there is a lack of interventions that promote self-management skills that prevent deterioration and improve health. The internet-based programme ‘Strategies for Empowering Activities in Everyday Life’ (SEE) supports people with long-term health conditions in developing self-management skills through knowledge, self-analysis and management strategies, promoting active and healthy daily life. This study protocol aims to evaluate the feasibility of SEE 2.0 and the study design.

**Methods and analysis:**

This feasibility study employs a pretest and post-test design with no control group, embedded within a mixed-method approach. The participants will include 30–40 clients, occupational therapists and managers involved in delivering SEE 2.0 in primary healthcare and hospital-based services. Data will be collected through assessments and forms at baseline, 4 months and 12 months after inclusion. Additionally, qualitative methods will be used to enhance the understanding of participants’ experiences.

**Ethics and dissemination:**

The study was approved by the Swedish Ethical Review Authority. The results will inform the refinement of SEE 2.0 and guide decisions on whether to proceed with planning a full-scale evaluation to assess the intervention’s effect. The results will be published in peer-reviewed scientific journals on websites and presented at conferences and seminars to reach various user groups.

**Trial registration number:**

NCT06484322.

**STRENGTHS AND LIMITATIONS OF THIS STUDY**

- The intervention will leverage therapeutic mechanisms of the internet-based format to promote clients’ development of self-management in activities of everyday life.
- The combination of quantitative and qualitative data will provide a comprehensive assessment of the feasibility of the intervention and the study design.
- This study adopts a pragmatic approach, which, while challenging, will enhance the clinical applicability of the results.
- The small study sample without a control group will limit the ability to generalise potential effects.

## Introduction

 Establishing proactive interventions for people with chronic diseases and long-term disorders is a priority in Sweden and internationally and has the potential to use a person’s own resources to prevent deterioration and promote health.^1^,^3^ Many people may need early support to become proactive and develop self-management skills. For example, to promote a healthy balance of their engagement in various activities across different places and social contexts, aligned with what they want and need in their daily lives.^4^ Otherwise, and as a result of difficulties in managing their life situations, there is a risk that they will fall into a negative spiral of activity limitations and poor health.^4 5^ Thus, to prevent such limitations, it is crucial that healthcare provides early support to people with chronic diseases and long-term disorders across the different settings in which they receive care. This includes primary care as well as hospital-based outpatient and inpatient services. However, proactive interventions that support self-management are currently insufficient.^4^,^7^

Chronic disease encompasses long-term or lifelong health conditions that require constant attention and may also limit activities of everyday life.^8^ Long-term disorders are often of unclear origin and typically involve a combination of factors that affect everyday life, manifested primarily as symptoms of a behavioural nature.^9^ Some chronic diseases and long-term disorders have only a minimal impact and can often be effectively managed with medical and lifestyle-related measures. Others, such as rheumatological, neurological, cardiopulmonary, musculoskeletal and stress-related conditions, significantly affect people’s lives. The consequences of these long-term health conditions can be extensive, with reduced activity levels^10^,^12^ and varying degrees of difficulty in carrying out activities at home, in leisure time and at work.^11^,^15^ Living with altered activity levels can also affect sleep in many individuals.^16^ Unpredictable and fluctuating symptoms of long-term pain, stress and fatigue add to the complexity of everyday life and may necessitate extensive self-management.^617^,^19^ Fluctuations over days and longer periods pose challenges in developing effective management strategies.^17 18^ This may lead to difficulties in maintaining a healthy balance between different activities,^20^,^23^ which requires adapting the level and distribution of engagement when capacity varies.^18 24 25^ As a result, individuals across long-term health conditions often need to integrate a variety of management strategies to address these consequences in their everyday life.^612 17^,^19 26^

To meet these challenges, we have developed and initiated the evaluation of an internet-based occupational therapy intervention programme called ‘Strategies for Empowering activities in Everyday life’, SEE V.1.0.^427^,^31^ The programme supports people with long-term health conditions in developing self-management skills through knowledge, self-analysis and management strategies, promoting an active and healthy everyday life. SEE has three components: (1) an intervention programme including a web programme and materials for clients, (2) a web-based educational programme for the intervention providers—occupational therapists (OTs)—and (3) an intervention guide for OTs. Encouragingly, the results from previous studies of stroke clients in outpatient rehabilitation clinics^28 31^ indicate that SEE 1.0 facilitates self-management skills development and engagement in daily activities. Further, the OTs who delivered SEE demonstrated a transition towards an enhanced person-centred approach, facilitating the active role of clients during the intervention process.^29^ However, these studies^28 29 31^ revealed the continued need to evaluate the feasibility of the intervention programme across additional long-term health conditions and within a broader range of healthcare settings, following adjustments. Minor adjustments were needed to refine the intervention programme, therapist education programme and intervention guide. Furthermore, adjustments were needed to expand the target group, change the level of care/setting and revise recruitment procedures, introduce a new assessor-based outcome assessment and strengthen monitoring of adherence and implementation. After these changes were implemented, the first test of the updated version of SEE (V.2.0) was conducted in a case study.^32^ A feasibility study of SEE 2.0 in people with long-term health conditions within primary care and hospital-based services was then decided as the next step towards preparation for an evaluation trial. The present feasibility study is essential for identifying whether further adjustments of SEE 2.0 are needed and for assessing the research design for future large-scale studies. Moreover, even if an intervention proves to be feasible and effective, its adoption and integration can still be challenging. To overcome potential barriers to the integration of new interventions across healthcare settings, it is important to develop an understanding of relevant implementation aspects at an early stage.^33^,^35^

### Objectives

The project aims to evaluate the feasibility and implementation potential of SEE 2.0 for people with long-term health conditions across selected primary care and hospital-based services in Sweden. The following objectives were defined to assess SEE 2.0 and the study design:

- Evaluate participant recruitment and retention.
- Evaluate the assessments’ suitability to detect outcomes of SEE at 4 and 12 months after entry into the programme, as well as the acceptability of the assessments.
- Evaluate client participants’ acceptability and value of SEE.
- Explore client participants’ experiences with the SEE intervention process and its influence on their self-management, everyday life and health.
- Evaluate participant (client and OT) adherence to SEE.
- Explore OTs’ and managers’ experiences of feasibility, acceptability, value and implementation potential of deliver SEE across different healthcare settings.

## Methods and analysis

### Design

This feasibility study of SEE 2.0 uses a pretest and post-test design with no control group, embedded within a mixed-method approach.^3336^,^40^ The data collection comprises outcome assessments, qualitative semistructured interviews, focus group (FG) discussions and study-specific forms for descriptive data. The study adheres to the Medical Research Council guidelines^33 41 42^ for evaluating complex interventions, and the feasibility study protocol follows the ‘Standard Protocol Items: Recommendations for Intervention Trials’.^43 44^ The trial is registered at ClinicalTrials.gov (ID: NCT06484322). A logical model^45^ provides a simplified overview of how SEE, including its components, is designed and expected to work (online supplemental table S1).

### Study setting

SEE 2.0 will be provided across multiple levels of care in northern Sweden: through primary healthcare services, including three healthcare centres, and within hospital-based outpatient and inpatient services. The internet-based intervention, SEE 2.0 will be delivered to clients through the ‘Support and Treatment Platform’, an e-health service available via the Swedish Healthcare Guide (1177.se), along with video meetings with the deliverer/OT.

### Eligibility criteria

#### Clients

People aged 18–75 years diagnosed with or at risk of developing chronic disease or long-term disorders (collectively referred to as long-term health conditions) receiving care in the involved healthcare settings will be considered for inclusion. Additionally, inclusion requires that the person is at risk of, or experiences activity limitations, expresses a need to develop self-management skills in everyday life and is motivated and ready to make changes in activities of daily living.^46^ This will be ascertained through a set of yes/no screening questions. To complete the programme, access and ability to use a screen/computer, internet and e-identification are also required. The exclusion criteria include conditions that significantly hinder the implementation of written and oral advice (eg, mental or cognitive illness), substance abuse and need for palliative care.

#### Professionals

The recruitment pool will consist of OTs, their managers and other staff in the healthcare settings who have volunteered to deliver SEE 2.0. No specific inclusion or exclusion criteria will be applied.

### Intervention

#### Intervention programme SEE 2.0 for clients

The goal of SEE 2.0 is to foster a well-distributed and balanced level of engagement in various daily activities across different contexts, including places and social interactions.^4 32^ To promote an active everyday life, the programme empowers clients with long-term health conditions to learn, self-analyse and change activities in everyday life. By gaining insights into their own patterns and balance in activities, clients can develop self-initiated management strategies to promote their health and well-being. By subsequently paying attention to these strategies, they can initiate and identify what works well and take an active role in preventing and overcoming challenges sustainably over time.

The internet-based SEE 2.0 intervention is person-centred and evolves through several phases, each comprising one or more modules.^4 32^ Throughout the entire intervention process, an OT continuously supports clients’ self-directed activities and change process related to each module by providing written feedback (asynchronous) and guided dialogues (synchronous), both delivered online. In the first web module in the intervention process, clients are supported in exploring their motivation and readiness to implement changes in their everyday activities. This is followed by an in-depth dialogue with an OT to further address these issues. Subsequently, clients engage in self-analysis by completing assessments of their activity engagement and use of management strategies. Their findings are further explored in one to two meetings in guided dialogues with the OT. Next, clients complete six web modules one at a time, each including concise educational videos accompanied by self-analyses and reflective tasks. Their work across the modules is supported by written feedback from the OT and by three guided in-depth dialogues with the OT, held at selected points during the process. The six modules aim to enhance self-management by imparting knowledge about strategies and modifications for everyday activities that can prevent disability and promote health. When clients have completed all modules and dialogues with the OTs, together, they create an activity plan for how to change their everyday life, including specific goals and activity-based management strategies to achieve an active everyday life (approximately 3–4 weeks into the programme). Clients receive ongoing support from the OT when the plan is implemented, using a person-centred approach that includes follow-up meetings tailored to their needs. The intervention process is expected to last between 2 and 4 months, but clients’ process of change can continue also when the intervention has come to an end. The OT also contacts and coordinates with other professionals if needed to implement the plan. The intervention process is complemented by written client materials introducing the intervention process as well as summarising and repeating important content from the web modules.

In addition to SEE 2.0, all clients receive usual care when needed. Usual care involves meeting with a physician for medical treatment, sick leave and/or referrals to other healthcare professionals, such as physiotherapists, medical social workers, psychologists and psychotherapists.

#### Educational programme for training SEE 2.0 interventionists

Before being eligible to deliver SEE 2.0, OTs complete a web-based educational programme and familiarise themselves with the intervention guide to ensure adherence to the intervention. During an online session, researchers introduce OTs to the self-paced educational format and encourage them to note any questions or topics for discussion in a follow-up meeting after the training. The educational programme includes videos that cover the evidence, theories and models underpinning the SEE programme. Additionally, there are practical videos demonstrating how to effectively deliver SEE to clients. An online session also guides OTs on how to assign clients to SEE on the National Healthcare Platform. In addition to the videos, OTs learn to navigate the intervention guide, which provides a step-by-step instruction for each module, emphasising person-centredness and the importance of active client engagement. After completing the training, OTs participate in a follow-up meeting with researchers to address any questions that they may have and repeat practical tips to prepare them to deliver SEE to clients. Throughout the study, OTs can reach out to researchers for supervision and support in implementing SEE as needed.

### Participant timeline

The study timeline is illustrated in the online supplemental table S2. The enrolment of the client participants began in September 2024 and is expected to be completed by December 2026. The person-centred intervention process is anticipated to vary between 2 and 4 months. Clients’ change processes and subsequent improvements are expected to continue even after the interventions end. Consequently, to identify potential long-term outcomes, evaluations will be conducted 4 months and 12 months after the start of SEE 2.0.

### Sample size

The estimated number of client participants required to achieve the study objective is 30–40. This number is considered adequate to provide sufficient variation and to ensure valuable insights into the feasibility aspects assessed in this study.^37^ In feasibility studies, formal sample size calculations are rarely required, as statistical powering of future trials based on such studies is often unreliable.^37 39 40^ Instead, recommended practice is to recruit a sample that is large enough to capture variability in participant characteristics and to identify potential barriers to implementation. The chosen sample size of 30–40 participants is therefore deemed sufficient for these purposes while remaining feasible within the scope of the project.

### Recruitment

Potential clients can receive information in different ways from the involved healthcare settings. The healthcare staff can give a short oral briefing about the intervention programme and study during visits or telephone calls. Alternatively, information is provided via brochures and screens in the waiting rooms. Those who are interested in participation receive an information letter with complete details about the study. If they agree to participate, they send the informed consent (online supplemental material S3) to the researchers.

Once the researchers receive the consent form, potential participants are contacted by telephone. The content of the information letter is presented orally, and the potential participants are given the opportunity to ask questions to confirm their consent. They are then screened for the inclusion criteria, and if they meet the criteria, baseline assessments are initiated.

Personnel at the involved healthcare settings, including OTs, managers and other involved staff, receive oral and written information about the project before their written informed consent is sought. OTs and managers who are interested in being included in the study are asked if they would like to contribute, in addition to recruiting clients, by delivering SEE. Thus, SEE 2.0 will be delivered by OTs who want to deliver the new intervention and complete the educational programme.

### Data collection

An overview of the timeline of the various methods used to collect data from clients, OTs, managers and other staff is presented in online supplemental table S2. Outcome assessments will be conducted at baseline and at 4 and 12 months postintervention. Additionally, at 4 months, clients will rate the acceptability and value of SEE 2.0. Intervention adherence will be evaluated by forms and field notes throughout the intervention. Qualitative experiences of the programme will be gathered through semistructured interviews with clients and FG discussions with staff.

#### Enrolment and retention

The enrolment, inclusion, retention and drop-out rates of the participants will be measured by the researchers during the study period.

#### Suitability and acceptability of outcome assessments

To evaluate the assessments’ suitability for detecting the outcomes of SEE 2.0 and their acceptability, responses to the assessments and complete and incomplete assessments, including missing values, will be registered. The primary outcome is engagement in activities, and potential improvements will be assessed from different perspectives. Satisfaction with Daily Occupations-Occupational Balance (SDO-OB)^47 48^ is an interview-based questionnaire that, in 13 items, addresses the activity level (ranging from 0 to 13), satisfaction with daily activities (ranging from 13 to 91) and general balance in activities from a time allocation perspective (five response alternatives ranging from −2 to +2). The Occupational Balance Questionnaire (OBQ-11)^49^ is a self-report questionnaire assessing balance in activities consisting of 11 items, each rated on a 4-point Likert scale. The scores are summed to yield a total score ranging from 0 to 33 points. The Occupational Value Assessment with Predefined items (Oval-pd)^50 51^ is a self-report questionnaire that assesses the perception of occupational value. The Oval-pd includes 18 items rated on a 4-point Likert scale, with total scores ranging from 18 to 72.

The secondary outcomes focus on aspects such as work ability, health and symptoms. The Work Ability Index (WAI) assesses self-perceived work ability on a 10-point ordinal scale. It also uses a 5-point Likert scale to evaluate the physiological and psychological demands of work. A higher score signifies a higher level of perceived work ability.^52^ A question will be used to assess work ability and sick leave percentage. Three items from the Life Satisfaction Questionnaire (LiSat-11) will be used to assess satisfaction with life as a whole, physiological health and psychological health. These self-reports will be assessed using a 6-point Likert scale (1=very dissatisfied, 6=very satisfied).^53 54^ The EuroQol Visual Analogue Scale (EQ VAS)^55^ will be used to assess health-related quality of life. The EQ VAS is a vertical scale ranging from 0 to 100, with 0 representing the ‘worst possible health state’ and 100 representing the ‘best possible health state’. A mark on the scale represents an individual’s current overall health status based on his or her own perceptions. The RAND-36 health survey^56^ is a self-report questionnaire that assesses functioning and well-being across 36 items in eight dimensions, each ranging from 0 to 100.

To assess self-related belief in one’s ability to effectively deal with stressful situations, the General Self-Efficacy Scale (GSE-10) will be used. The GSE-10 consists of 10 items, each scored on a 4-point Likert scale. The total score ranges from 10 to 40, with specific cut-offs indicating different levels of self-efficacy.^57^ The Perceived Stress Scale (PSS-10)^58^ will measure self-reported stress perceptions over the past month with 10 items, which are summed to range from 0 to 40. The Mental Fatigue Scale (MFS)^59^ contains 15 items for self-reporting of affective, cognitive and sensory symptoms related to fatigue. The ratings for the items are summed, indicating the level of severity of the symptoms. All the instruments (SDO-13, OBQ11, Oval-pd, WAI, LiSat, EQ VAS, RAND-36, GSE-10, PSS-10, MFS) are reliable and valid, and higher scores on (all of) the assessments indicate greater/higher levels of experience with the assessed aspect. The self-reported assessments (OBQ11, Oval-pd, WAI, self-estimated work ability/sick leave, LiSat, EQ VAS, RAND-36, GSE-10, PSS and MFS) will be sent by mail with return envelopes for participants’ responses. Once the researcher receives the responses, a video meeting will be arranged to conduct the SDO interview. Data on sociodemographic and care received during the study period will also be collected.

#### Acceptability and value of SEE 2.0

Client acceptability of SEE 2.0 components and perceived value will be assessed using study-specific self-report forms.^31^ In conjunction with the forms, potential harm will be assessed with an open-ended written question. These forms will be included in the postal questionnaire sent out for the 4-month assessments.

#### Client experiences of SEE 2.0

Qualitative interviews will be conducted with clients receiving SEE 2.0 to explore their experiences with the SEE intervention process and its influence on self-management, everyday life and health. A purposeful selection of a heterogeneous sample^60^ of 6–12 clients will take place until saturation^61^ in the data is reached. Clients will be interviewed on two occasions: 1 month into the programme and after completion (4–5 months after the start). The qualitative, open, in-depth interviews^62^ will include questions addressing the clients’ experiences of how they are developing self-management during the intervention process and its meaning for activities in everyday life. The participants’ experience of whether the change process evolves, as well as their learning and adoption of self-analysis and management strategies, will also be a focus.

#### Intervention adherence

To detect adherence to SEE’s intervention process and guide (2.0), a study-specific registration form will be used for structured interviews with OTs at two points: when phase 4 is completed and when the intervention is completed. In addition, researchers will take continuous field notes on aspects of feasibility, including the adherence and implementation observed during meetings with participants (both clients and OTs).

#### OTs’ experiences with SEE 2.0

FG discussions with periodic recurring discussions^63 64^ will take place with the OTs who deliver SEE 2.0 during the study period. The key questions in the discussions will focus on the feasibility, acceptability, value and implementation of SEE, including how the educational programme and the intervention guide support the delivery of the programme.

#### Professionals’ experiences with SEE 2.0

FG discussions^63 64^ will be conducted with various groups of participants who have used SEE to explore the feasibility and implementation of SEE 2.0. The FGs will take place in 2–4 groups with 3–5 participants each to collect data on SEE feasibility, acceptability, value and implementation. The key questions will also focus on opportunities and obstacles for integrating SEE into various healthcare settings, referral processes and different care pathways, as well as the potential of transferability to other contexts.

### Data management

Data will be stored securely with limited access in accordance with regulations and guidelines, as described in the data management plan. Participants will be assigned a code number, which will be used to pseudonymise all the research data. The list of participants’ code numbers will be stored securely in a locked cabinet, separately from the data and consent forms. The data from the assessments and study-specific forms will be transferred to a computer file for calculation. The qualitative interviews and FGs will be recorded on an audio file and subsequently transcribed pseudonymically to word files. Creation of the files will involve processes to check and promote data quality. When the data are transferred to computer files, access will require multifactor authentication with passwords. The data will be stored on LTUdrive, a storage service provided by Luleå University of Technology (LTU) in Sweden. This service meets the requirements for secure data storage, sharing and protection in accordance with research ethics and the General Data Protection Regulation (GDPR).^65^ After the project is completed, read-me files that enable the reuse of data, along with all related materials, will be securely stored long-term in the central archive at LTU.

### Data analysis

The statistical analysis will assess the suitability of the assessments in detecting outcomes of SEE 2.0 at 4 and 12 months after entry into the programme. In this study, descriptive statistics and non-parametric tests will be used to identify patterns of changes and compare outcomes on respective outcome assessments over time, from baseline to 4 and 12 months. The intention-to-treat approach will be applied, and per-protocol analyses will be conducted if necessary. A gender perspective will be considered in the data analysis to identify potential differences and similarities in outcomes. Sociodemographic characteristics in relation to outcomes will be analysed descriptively. The significance level will be set at p<0.05 for all the statistical tests. All the data from the enrolment process, the number of complete or incomplete assessments, study-specific forms for adherence, acceptability, value, potential harms and field notes will be compiled descriptively^38 66^ by analysis of content and descriptive statistics. The qualitative interviews with the clients will be analysed using the constant comparative method of grounded theory,^61^ as this method is suitable for identifying patterns in interactions, strategies and processes such as intervention processes. The FG discussions will be analysed with FG analysis^63 64^ to identify themes in the collective experiences.

## Patient and public involvement

The intervention programme SEE is developed together with users based on their needs,^4^ with their perspective being central. SEE is designed to meet clients’ need for support in developing self-management strategies to manage activities of everyday life by improving access to care through person-centred internet-based interventions. The choice of outcome assessments is informed by their priorities. SEE has thus far been evaluated with contributions from users and other concerned parties.^27^,^2931 32^ This protocol is also designed to incorporate the experiences of clients, OTs, managers and other staff. Researchers will consistently update users on the project’s progress and actively involve them in discussions about various research steps and dissemination methods.

## Ethics and dissemination

The project has been approved by the Swedish Ethical Review Authority (EPM Dnr: 2023-08032-01, 2024-042277-02, 2025-00635-02, 2025-04193-02) and will be conducted in accordance with the Declaration of Helsinki,^67^ the Swedish law of ethical research on humans^68^ and the GDPR.^65^ All participants will receive information about what participation in the research means both orally and in writing and can ask questions before they sign the written informed consent form and send it to the researchers. This includes information about the potential benefits or risks of participation and the right to withdraw their participation in the study at any time without explanation. They will also be informed that the data will be treated as confidential. Given the small sample size from a local perspective, the amount of personal information will be limited to ensure that participants’ identities cannot be derived from study presentations as in scientific papers.

During the implementation of the internet-based intervention SEE 2.0, patient safety and the protection of sensitive data are ensured by using the support and treatment platform on Sweden’s 1177 National Healthcare Guide. Logging in to the platform is performed via national e-identification. Based on person-centredness, clients can choose whether to carry out the guiding dialogues that support their change process digitally or via personal meetings at the healthcare settings. For digital meetings, secure forums recommended by the organisation will be used. The researchers will not have access to 1177 or medical records.

The results will be presented at conferences that attract researchers, professionals and the general public. Particular emphasis will be placed on reaching out to interest groups (such as patient and professional associations). The studies will be published in international scientific journals, and the researchers will adhere to established authorship guidelines. Popular science summaries of the results will also be created for various audiences and shared on the LTU website and social media.

## Discussion

This study aims to further assess both SEE 2.0 and the study design to determine the feasibility prior to proceeding to a full-scale evaluation in the next step of our research. A major strength of the design is that it is informed by results from our previous research,^27 28 31^ addressing remaining uncertainties about programme feasibility and by our original intentions^4^ to evaluate the use of SEE in a broader client population and at various levels of care. Another advantage is its pragmatic approach,^69^ with SEE being delivered by OTs in various standard healthcare settings. The inclusion criteria for clients are broad enough to reflect the typical pool of clients with limitations in activities of everyday life. Additionally, multiple healthcare settings are involved in recruiting clients to ensure the representation of individuals with different diagnoses from both rural and urban areas with diverse socioeconomic backgrounds. At the same time, a potential limitation is that delivering interventions in standard healthcare settings may be influenced by organisational changes and adherence difficulties to the delivery of the programme. These factors could affect the consistency of implementation. However, such challenges also provide valuable information on the feasibility, acceptability and implementation of SEE in routine practice from the perspectives of different user groups. Furthermore, this pragmatic approach increases the clinical applicability of the results across different user groups and settings, while highlighting the adjustments needed to improve both the intervention and the study design.

Another strength of SEE 2.0 is its innovative character. It is unique in that it is not standard practice to deliver complete occupational therapy intervention processes via the web, and currently no other occupational therapy intervention is available through Sweden’s National Healthcare Platform. Thus, the development and testing of SEE represent an important contribution to the digitalisation of occupational therapy. A further advantage is that the internet-based format improves access to person-centred support and empowers clients, which may lead to more sustainable health outcomes over time. By including the flipped classroom approach^70^ in the programme theory,^4 30^ SEE also introduces therapeutic mechanisms that are not available in traditional in-place rehabilitation.^28^ A possible limitation, however, is that digital delivery may not suit all clients, particularly those with limited digital literacy, cognitive challenges or poor access to technology. Conversely, it may be particularly suitable for clients living at a distance from care facilities or with reduced capacity to travel. SEE’s internet-based format, which combines clients’ own preparation work with guided dialogues with an OT, is designed to improve clients’ self-management and promote sustainable health outcomes. A strength of this approach is that it fosters self-analysis and activity-focused management strategies, supporting a healthy balance of engagement in everyday activities when capacity changes. The knowledge gained will inform the design of future internet-based interventions and their implementation in practice.

## Supplementary material

10.1136/bmjopen-2025-102026 online supplemental file 1

10.1136/bmjopen-2025-102026 online supplemental file 2

10.1136/bmjopen-2025-102026 online supplemental file 3

## References

[1] World Health Organisation (WHO) (2013). Global action plan for the prevention and control of NCDs 2013–2020, Geneva.

[2] Regeringskansliet (2014). Nationell strategi för att förebygga och behandla kroniska sjukdomar. 2014-2017. [A national strategy for prevention and treatment of chronic diseases. 2014-2017].

[3] Nergårdh A, Andersson L, Eriksson J (2018). God och nära vård–En primärvårdsreform. [personcentered and integrated care- a primary health care reform].

[4] Larsson Lund M, Månsson Lexell E, Nyman A (2022). Optimising the development of sustainable internet-based occupational therapy interventions: Important key actions and perspectives to consider. Scand J Occup Ther.

[5] Riegel B, Westland H, Iovino P (2021). Characteristics of self-care interventions for patients with a chronic condition: A scoping review. Int J Nurs Stud.

[6] Warner G, Packer TL, Kervin E (2019). A systematic review examining whether community-based self-management programs for older adults with chronic conditions actively engage participants and teach them patient-oriented self-management strategies. Patient Educ Couns.

[7] (2018). Myndigheten för vård- och omsorgsanalys. Stöd på vägen. En uppföljning av satsningen på att förbättra vården för personer med kroniska sjukdomar. Rapport 2018:13. [Support along the way. A follow-up of the initiative to improve care for peoplewith chronic diseases].

[8] Goodman RA, Posner SF, Huang ES (2013). Defining and Measuring Chronic Conditions: Imperatives for Research, Policy, Program, and Practice. Prev Chronic Dis.

[9] Spitzer RL, Endicott J (2018). Medical and mental disorder: Proposed definition and criteria. Annales Médico-Psychologiques, Revue Psychiatrique.

[10] Harker J, Reid KJ, Bekkering GE (2012). Epidemiology of chronic pain in denmark and sweden. Pain Res Treat.

[11] Nielsen KT, Klokker L, Wæhrens EE (2021). Self-reported quality of activities of daily living task performance in four diagnostic groups with chronic conditions. Int J Ther Rehabil.

[12] Klinedinst TC, Terhorst L, Rodakowski J (2022). Multimorbidity groups based on numbers of chronic conditions are associated with daily activity. Chronic Illn.

[13] Dueñas M, Salazar A, de Sola H (2020). Limitations in Activities of Daily Living in People With Chronic Pain: Identification of Groups Using Clusters Analysis. Pain Pract.

[14] Audulv Å, Packer T, Versnel J (2014). Identifying gaps in knowledge: A map of the qualitative literature concerning life with a neurological condition. Chronic Illn.

[15] Daniel K, Wolfe CDA, Busch MA (2009). What are the social consequences of stroke for working-aged adults? A systematic review. Stroke.

[16] Koyanagi A, Garin N, Olaya B (2014). Chronic conditions and sleep problems among adults aged 50 years or over in nine countries: a multi-country study. PLoS ONE.

[17] Audulv A, Hutchinson S, Warner G (2021). Managing everyday life: Self-management strategies people use to live well with neurological conditions. Patient Educ Couns.

[18] Audulv Å, Ghahari S, Kephart G (2019). The Taxonomy of Everyday Self-management Strategies (TEDSS): A framework derived from the literature and refined using empirical data. Patient Educ Couns.

[19] Andersen LN, Kohberg M, Juul-Kristensen B (2014). Psychosocial aspects of everyday life with chronic musculoskeletal pain: A systematic review. Scand J Pain.

[20] Ortiz-Rubio A, Cabrera-Martos I, Haro-Piedra E (2022). Exploring perceived occupational balance in women with fibromyalgia. A descriptive study. Scand J Occup Ther.

[21] Kassberg AC, Nyman A, Larsson Lund M (2021). Perceived occupational balance in people with stroke. Disabil Rehabil.

[22] Wagman P, Ahlstrand I, Björk M (2020). Occupational balance and its association with life satisfaction in men and women with rheumatoid arthritis. Musculoskeletal Care.

[23] Nyman A, Kassberg A-C, Larsson Lund M (2021). Perceived occupational value: Relationships to occupational balance, lifesatisafaction and health in people with stroke. Scand J Occup Ther.

[24] Olofsson A, Nyman A, Larsson Lund M (2017). Occupations outside the home: Experiences of people with acquired brain injury. Br J Occup Ther.

[25] Eklund M, Orban K, Argentzell E (2017). The linkage between patterns of daily occupations and occupational balance: Applications within occupational science and occupational therapy practice. Scand J Occup Ther.

[26] Nygard L, Ryd C, Astell A (2022). Self-initiated management approaches in everyday occupations used by people with acquired cognitive impairment. Scand J Occup Ther.

[27] Barcheus IM, Ranner M, Nyman A (2023). Developing and testing the feasibility of a new internet-based intervention-A case study of people with stroke and occupational therapists. PLoS ONE.

[28] Barcheus IM, Ranner M, Mansson Lexell E (2024). The Internet-Based Intervention Strategies for Empowering Activities in Everyday Life: Qualitative Study of Experiences of Clients With Stroke. JMIR Form Res.

[29] Barcheus IM, Ranner M, Mansson Lexell E (2024). Occupational therapists’ experiences of using a new internet-based intervention - a focus group study. Scand J Occup Ther.

[30] Larsson-Lund M, Mansson Lexell E, Nyman A (2021). Strategies for Empowering activities in Everyday life (SEE 1.0): study protocol for a feasibility study of an Internet-based occupational therapy intervention for people with stroke. Pilot Feasibility Stud.

[31] Larsson-Lund M, Barcheus I-M, Ranner M (2024). A feasibility study of the internet-based intervention “Strategies for Empowering activities in Everyday life” (SEE 1.0) applied for people with stroke. BMC Health Serv Res.

[32] Lindberg M, Ranner M, Jacobsson L (2024). Can the internet based intervention “strategies for empowering activities in everyday life” support people with cognitive difficulties to self-manage digital work and everyday life?. Scand J Occup Ther.

[33] Skivington K, Matthews L, Simpson SA (2021). A new framework for developing and evaluating complex interventions: update of Medical Research Council guidance. BMJ.

[34] Hawe P (2015). Lessons from complex interventions to improve health. Annu Rev Public Health.

[35] Colquhoun HL, Squires JE, Kolehmainen N (2017). Methods for designing interventions to change healthcare professionals’ behaviour: a systematic review. Implementation Sci.

[36] O’Cathain A, Hoddinott P, Lewin S (2015). Maximising the impact of qualitative research in feasibility studies for randomised controlled trials: guidance for researchers. Pilot Feasibility Stud.

[37] Thabane L, Ma J, Chu R (2010). A tutorial on pilot studies: the what, why and how. BMC Med Res Methodol.

[38] Richards DA, Hallberg IR (2015). Complex interventions in health: an overview of research methods.

[39] Hallingberg B, Turley R, Segrott J (2018). Exploratory studies to decide whether and how to proceed with full-scale evaluations of public health interventions: a systematic review of guidance. Pilot Feasibility Stud.

[40] Moore GF, Evans RE, Hawkins J (2019). From complex social interventions to interventions in complex social systems: Future directions and unresolved questions for intervention development and evaluation. Evaluation (Lond).

[41] Craig P, Dieppe P, Macintyre S (2008). Developing and evaluating complex interventions: the new Medical Research Council guidance. BMJ.

[42] Moore GF, Audrey S, Barker M (2015). Process evaluation of complex interventions: Medical Research Council guidance. BMJ.

[43] Chan A-W, Boutron I, Hopewell S (2025). SPIRIT 2025 statement: updated guideline for protocols of randomised trials. The Lancet.

[44] Hróbjartsson A, Boutron I, Hopewell S (2025). SPIRIT 2025 explanation and elaboration: updated guideline for protocols of randomised trials. BMJ.

[45] Mills T, Lawton R, Sheard L (2019). Advancing complexity science in healthcare research: the logic of logic models. BMC Med Res Methodol.

[46] Prochaska JO, DiClemente CC, Nrcross JC, Goldfried MR (2005). Handbook of psychotherapy integration.

[47] Eklund M, Argentzell E (2016). Perception of occupational balance by people with mental illness: A new methodology. Scand J Occup Ther.

[48] Wästberg BA, Persson EB, Eklund M (2016). The Satisfaction with Daily Occupations (SDO-13) Scale: Psychometric Properties among Clients in Primary Care in Sweden. Occup Ther Health Care.

[49] Håkansson C, Wagman P, Hagell P (2020). Construct validity of a revised version of the Occupational Balance Questionnaire. Scand J Occup Ther.

[50] Eakman AM, Eklund M (2011). Reliability and structural validity of an assessment of occupational value. Scand J Occup Ther.

[51] Eklund M, Erlandsson LK, Persson D (2009). Rasch analysis of an instrument for measuring occupational value: Implications for theory and practice. Scand J Occup Ther.

[52] de Zwart BCH (2002). Test-retest reliability of the Work Ability Index questionnaire. Occup Med.

[53] Fugl-Meyer AR, Melin R, Fugl-Meyer KS (2002). Life satisfaction in 18- to 64-year-old Swedes: in relation to gender, age, partner and immigrant status. J Rehabil Med.

[54] Melin R, Fugl-Meyer KS, Fugl-Meyer AR (2003). Life satisfaction in 18- to 64-year-old Swedes: in relation to education, employment situation, health and physical activity. J Rehabil Med.

[55] Devlin NJ, Brooks R (2017). EQ-5D and the EuroQol Group: Past, Present and Future. Appl Health Econ Health Policy.

[56] Orwelius L, Nilsson M, Nilsson E (2018). The Swedish RAND-36 Health Survey - reliability and responsiveness assessed in patient populations using Svensson’s method for paired ordinal data. *J Patient Rep Outcomes*.

[57] Löve J, Moore CD, Hensing G (2012). Validation of the Swedish translation of the General Self-Efficacy scale. Qual Life Res.

[58] Nordin M, Nordin S (2013). Psychometric evaluation and normative data of the Swedish version of the 10-item perceived stress scale. Scand J Psychol.

[59] Johansson B, Ronnback L (2014). Evaluation of the Mental Fatigue Scale and its relation to Cognitive and Emotional Functioning after Traumatic Brain Injury or Stroke. Int J Phys Med Rehabil.

[60] Holloway I, Galvin K (2024). Qualitative research in nursing and healthcare.

[61] Charmaz K (2006). Constructing grounded theory: a practical guide through qualitative analysis.

[62] Kvale S, Brinkmann S (2014). Den kvalitativa forskningsintervjun. [The qualitative research interview].

[63] Dahlin-Ivanoff S, Holmgren K (2017). Fokusgrupper. [Focusgroups].

[64] Krueger RA, Casey MA (2014). Focus groups: a practical guide for applied research.

[65] European Union, European Data Protection Board General data protection regulation (GDPR)2016/679. https://ec.europa.eu/info/law/law-topic/data-protection_sv.

[66] Patton MQ (2015). Qualitative evaluation and research methods.

[67] World Medical Association Declaration of Helsinki. Ethical principles for medical research involving human subjects. https://www.wma.net/what-we-do/medical-ethics/declaration-of-helsinki/.

[68] Lag (2003:460) om etikprövning av forskning som avser människor [law (Sfs 2003:460) concerning the ethical review of research involving humans]. https://www.riksdagen.se/sv/dokument-lagar/dokument/svensk-forfattningssamling/lag-2003460-om-etikprovning-av-forskning-som_sfs-2003-460.

[69] Williams HC, Burden-Teh E, Nunn AJ (2015). What is a pragmatic clinical trial?. J Invest Dermatol.

[70] Abeysekera L, Dawson P (2015). Motivation and cognitive load in the flipped classroom: definition, rationale and a call for research. Higher Educ Res Dev.

