# Supplementary material for: Feasibility study of the internet-based intervention ‘Strategies for Empowering activities in Everyday life’ (SEE 2.0) for use by people with chronic diseases and long-term disorders in healthcare: a study protocol
Source: BMJ Open. 2025 Oct 21;15(10):e102026. doi: 10.1136/bmjopen-2025-102026 (PMC12548600; doi:10.1136/bmjopen-2025-102026)
Supplement: online supplemental file 1 [file bmjopen-15-10-s001.pdf]

Supplementary Table S1. Logic model of Strategies for Empowering activities in Everyday life, SEE 2.0

**Needs addressed by SEE**	**Educational program for SEE and referral routines to identify clients in need of SEE 2.0**	**Application of SEE 2.0 to clients**	**Therapeutic mechanism of change**	**Outcomes**
→	→	→	→	→
Context: Client and societal needs	Education and training of interventionists (organisational cointervention)	Clients' intervention process in five phases<br>(core intervention)	Program theory and related expected changes	Evaluation methods
Interventions that prevent deterioration, inactivity and ill health related to a chronic disease or long-term disorder<br><br>Interventions that support self-management in activities of everyday life and health<br><br>Interventions facilitating clients' ability to self-analyse and develop strategies that are flexible and sustainable and support an active everyday life over time<br><br>Need to improve access to interventions by flexible internet-based solutions	Educational program to train OTs in delivering SEE adherent and consistently including SEE's<br>- background, aim and implementation of the intervention process<br>- program theory and how it informs the intervention process and its therapeutic mechanism for change<br><br>Educational session in real time on how to manage SEE on the health care platform<br><br>Intervention guide for OTs with step-by-step guidance for delivering the SEE intervention process, including OTs' approach and acting	Phase 1. Modules for introducing SEE and evaluating motivation for changes in everyday life.<br>Phase 2. Modules for evaluating changes in activities in everyday life and management strategies.<br>Phase 3. Modules for self-analysis of activities in everyday life and management strategies in relation to health<br>Phase 4. Establishing an activity plan for changes towards a sustainable active everyday life.<br>Phase 5. Implementing the activity plan for change	*The programme theory's* core is models for how people's activities, behaviours and doing in everyday life influence well-being and health and can be used as a therapeutic means for change<br>The programme theory is further informed by person-centredness, self-management theory, prevention and rehabilitation, motivational interviewing, motivation theory for behavioural change and principles for flipped classroom<br><br>*Expected behavioural changes in clients:*	*Clients:*<br>-Satisfaction with daily occupations<br>-Occupational balance<br>-Occupational value<br>-Work ability and sick leave<br>-Life satisfaction and health<br>-Self-efficacy<br>-Stress<br>-Fatigue<br>-Consumption of care<br>-Study-specific forms about the acceptability of SEE components and value of SEE<br>-Field notes about the feasibility of SEE for each client and OT<br>-Repeated interviews on experiences of the change process, adopting of self-management and meaning

Need to implement feasible new evidence-based interventions and related new ways of organising and working that are sustainable in health care organisations<br><br>Need to develop professional roles and competences (knowledge, skills, attitudes) to enhance proactive internet-based person-centred care	Information about SEE to staff<br><br>Development of referral routines for clients in need of SEE		Increased knowledge, confidence and abilities to self- analyse and adopt management strategies in activities supporting an active everyday life with well-being and health.<br><br>*Expected changes in the competences of OTs:*<br>Increased competence (knowledge, skills and approaches) in delivering an internet- based intervention focusing on supporting clients' proactive self-management of activities in everyday life and, also, that the competence is also transferred to other clients outside the project, both during and after the project<br><br>*Expected changes in the organisation/setting:*<br><br>Increased insight into how proactive referral processes and internet-based interventions can be implemented in a sustainable way	-Recruitment, enrolment, allocation, retention and drop-out rates<br><br>*OTs:*<br>-Study-specific registration form for structured interviews on the feasibility of and adherence to SEE's intervention guide<br>- Field notes about the feasibility of SEE for each client and OT<br>-Focus group discussion about feasibility, acceptability, value, implementation and transferability of SEE<br><br>*Organisation level (managers, professionals and client representatives):*<br>Focus group discussion about feasibility, acceptability, value, implementation and transferability of SEE and of the referral process

			Increased insight into which clients are suitable for SEE or other OT treatment will contribute to sustainable use of OT resources and cost-effectiveness	

OT: Occupational therapist
