# Supplementary material for: Feasibility study of the internet-based intervention ‘Strategies for Empowering activities in Everyday life’ (SEE 2.0) for use by people with chronic diseases and long-term disorders in healthcare: a study protocol
Source: BMJ Open. 2025 Oct 21;15(10):e102026. doi: 10.1136/bmjopen-2025-102026 (PMC12548600; doi:10.1136/bmjopen-2025-102026)
Supplement: online supplemental file 2 [file bmjopen-15-10-s002.pdf]

Supplementary Table S2: Study timeline and data collection

		Trial period					
**Time point**		Baseline<br>0 month	1<br>weeks	4<br>weeks	4<br>months	12<br>months	After last client completed SEE
**Enrolment**							
Eligibility screen	x						
Informed consent	x						
Allocation							
**Client intervention**							
SEE		→	→	→	→		
**Assessments, clients**							
Demographics		x					
Primary outcomes							
SBO-OB		x			x	X	
OBQ11		x			x	X	
Oval-pd		x			x	X	
Secondary outcomes							
Actual work ability and sick leave		x			x	X	
WAI		x			x	X	
Lisat-11		x			x	X	
EQ VAS		x			x	X	
RAND-36		x			x	X	
GSE-10		x			x	X	
PSS-10		x			x	X	
MFS		x			x	X	
Retention and dropout rates		→	→	→	→	→	
Care consumption/received care		→	→	→	→	→	

Acceptability and value of SEE, study-specific forms					x		
Feasibility and adherence of SEE, researchers' field notes		→	→	→	→		
Experiences of SEE, qualitative interviews				x	x		
**Assessments, occupational therapists**							
Feasibility and adherence, study-specific registration forms				x	x		
Feasibility and adherence of SEE, researchers' field notes		→	→	→	→		
Experiences of SEE, focus group discussions, periodically repeated during study period							x
**Assessments, professionals**							
Experiences of SEE, focus group discussions							x

SBO-OB: Satisfaction with Daily Occupations-Occupational Balance; OBQ-11: Occupational Balance Questionnaire; Oval-pd: Occupational Value Assessment with Predefined Items; WAI: Work Ability Index; Lisat-11: Life Satisfaction Questionnaire; EQ VAS: EuroQol-visual analogue scale; RAND-36: RAND 36-item Health Survey; GSE-10: General Self-Efficacy Scale; PSS-10: Perceived Stress Scale; MFS: Mental Fatigue Scale.
