# Supplementary material for: Feasibility study of the internet-based intervention ‘Strategies for Empowering activities in Everyday life’ (SEE 2.0) for use by people with chronic diseases and long-term disorders in healthcare: a study protocol
Source: BMJ Open. 2025 Oct 21;15(10):e102026. doi: 10.1136/bmjopen-2025-102026 (PMC12548600; doi:10.1136/bmjopen-2025-102026)
Supplement: online supplemental file 3 [file bmjopen-15-10-s003.pdf]

Jag har fått muntlig och/eller skriftlig information om projektet och har haft möjlighet att ställa frågor. Jag får behålla den skriftliga informationen.

- Jag samtycker till att delta i projektet: **Utvärdering av ett nytt internetbaserat behandlingsprogram "Strategier för ett aktivt vardagsliv".**

Plats och datum	Underskrift
	Namnförtydligande

Mina kontaktuppgifter:

Telefon……………………………………

E-post……………………………………………………………………………………

Adress……

Vänligen, sänd in samtyckestalongen i bifogat svarskuvert så kommer vi att kontakta dig direkt!
