# Supplementary material for: Feasibility study of the internet-based intervention ‘Strategies for Empowering activities in Everyday life’ (SEE 2.0) for use by people with chronic diseases and long-term disorders in healthcare: a study protocol
Source: BMJ Open. 2025 Oct 21;15(10):e102026. doi: 10.1136/bmjopen-2025-102026 (PMC12548600; doi:10.1136/bmjopen-2025-102026)
Supplement: Supplementary file 4 [file bmjopen-2025-102026.reviewer_comments.pdf]

PEER REVIEW HISTORY

BMJ Open publishes all reviews undertaken for accepted manuscripts. Reviewers are asked to complete a checklist review form and are provided with free text boxes to elaborate on their assessment. These free text comments are reproduced below.

ARTICLE DETAILS

Title (Provisional)

Study protocol for a feasibility study of the internet-based intervention "Strategies for Empowering activities in Everyday life" (SEE 2.0) for use by people with chronic diseases and long-term disorders in health care

Authors

Larsson-Lund, Maria; Karlsson Sundbaum, Johanna; Mansson Lexell, Eva; Spinord, Linda

---

VERSION 1 - REVIEW

**Reviewer**	**1**
**Name**	**Salzmann, Stefan**
**Affiliation**	**Philipps University of Marburg, Clinical Psychology and Psychotherapy**
**Date**	**17-Sep-2025**
**COI**	**None**

This study is testing a new online program called Strategies for Empowering Activities in Everyday Life (SEE 2.0). The program is designed to help people with long-term health problems manage their daily activities better and improve their well-being. Through online lessons, self-reflection tasks, and regular support from occupational therapists, participants learn how to balance their activities and develop strategies that make everyday life easier and healthier. The study will follow about 30–40 people in Swedish primary health care to see if the program is practical, easy to use, and valuable for both patients and health professionals. The results will guide improvements to the program and decide if it should be tested in a larger study. In general, increasing patients' activity levels is an important goal and thus the SEE intervention seems relevant. This is an interesting study. However, from my perspective, the authors could increase the clarity of the manuscript by being more specific and provide more details and examples on the study. It may further be helpful to think critically about the sequence of parts in the manuscript to make it easier for readers to understand the program. Please find below some more details:

Introduction

- The authors may provide examples of diseases and disorders

- It is somewhat unlcear to me how to identify people who are in need of this approach. Ist he approach applicable to everybody?

- „For people who are living with such diseases, experiencing stress, anxiety, pain and fatigue makes life" – It seems that there is missing a word.

- The following sentence is difficult to understand: „However, these qualitative studies along with a feasibility study, revealed the continued need to evaluate the feasibility of SEE." Why is a feasibility study necessary when there is already a feasibility study? Or does the first mentioning of the feasibility study refer to predecessor intervention (SEE 1.0)?

- The authors may consider of referring to SEE 2.0 throughout the manuscript when referring to the current study to increase clarity.

Methods

- Table 1: the abbreviation HCC should be expalined

- Inclusion criteria: „Additionally, the person should experience activity limitations; have a need to develop self-management skills in everyday life; and be motivated and ready to make changes to his or her activities of daily living" -> how will these criteria be assessed? Do these criteria refer to specific assessments with cut-off scores?

- The authors may provide more details on how the interaction between the OTs and clients work at each stage. Via telephone/video-conferencing/in person? The authors may further explain why the interactions seem to change.

- „The enrolment of the client participants began in September 2024 and is expected to be completed by 2026" -> When in 2026?

- „The justification of the number is based on that the sample size of that number will be sufficiently representative of the target population 37," -> the authors may provide more details on this claim

- Reagarding the qualitative analysis: Do interview guides already exist?

- The authors may consider moving the paragraph on „recruiting" before the intervention since this may help the reader bette runderstand the process of the study.

VERSION 1 - AUTHOR RESPONSE

Introduction:

Comment: The authors may provide examples of diseases and disorders.

Reply: We have added examples of rheumatologic, neurologic, cardiopulmonary, musculoskeletal, and stress-related conditions to clarify the target population.

Comment: It is somewhat unclear how to identify people who are in need of this approach. Is the approach applicable to everybody?

Reply: We clarified that SEE 2.0 is intended for individuals with activity limitations or at risk thereof, with a need to develop self-management skills, not for the general population.

Comment: The sentence 'For people who are living with such diseases, experiencing stress, anxiety, pain and fatigue makes life' seems incomplete.

Reply: We revised the end of the paragraph where the sentence was located, ensuring clarity and grammatical accuracy.

Comment: The following sentence is difficult to understand: 'However, these qualitative studies along with a feasibility study, revealed the continued need...'

Reply: We revised the text to clarify that the earlier feasibility study referred to SEE 1.0, while the current study tests SEE 2.0.

Comment: The authors may consider referring to SEE 2.0 throughout the manuscript to increase clarity.

Reply: We now consistently use SEE 2.0 when referring to the current intervention.

Methods:

Comment: Table 1: the abbreviation HCC should be explained.

Reply: HCC is deleted from the table as it was not needed.

Comment: Inclusion criteria: How will these criteria be assessed? Do they refer to specific assessments with cut-off scores?

Reply: We clarified that inclusion criteria are assessed via yes/no screening questions.

Comment: More details are needed on how the interaction between OTs and clients works at each stage. Why do the interactions change?

Reply: We specified that interactions are conducted via online video meetings, with written feedback provided digitally, and explained the use of combination asynchronous and synchronous modes.

Comment: 'The enrolment... expected to be completed by 2026' – When in 2026?

Reply: We specified that enrolment is expected to be completed by December 2026.

Comment: 'The justification of the number...' – more details needed.

Reply: We expanded the explanation, clarifying that 30–40 participants is sufficient to capture variation and provide feasibility insights, based on recommendations for feasibility/ pilot studies.

Comment: Regarding the qualitative analysis: Do interview guides already exist?

Reply: We have clarified the planned content of the interview guides. The guides are presently in Swedish and will be refined and finalized at a later stage of the project.

Comment: Consider moving the paragraph on 'recruiting' before the intervention description.

Reply: We maintained the SPIRIT-recommended order, as we find it logical to present the recruitment description in close connection with the sample size.
